# How managed a market? Modes of commissioning in England and Germany

**DOI:** 10.1186/1472-6963-13-S1-S8

**Published:** 2013-05-24

**Authors:** Rod Sheaff, Naomi Chambers, Nigel Charles, Mark Exworthy, Ann Mahon, Richard Byng, Russell Mannion

**Affiliations:** 1 University of Plymouth, UK; 2 Manchester Business School, UK; 3 RHUL, London, UK; 4 Plymouth University Peninsula Schools of Medicine and Dentistry, UK; 5 University of Birmingham, UK

## Abstract

**Background:**

In quasi-markets governance over healthcare providers is mediated by commissioners. Different commissioners apply different combinations of six methods of control ('media of power') for exercising governance: managerial performance, negotiation, discursive control, incentives, competition and juridical control. This paper compares how English and German healthcare commissioners do so.

**Methods:**

Systematic comparison of observational national-level case studies in terms of six media of power, using data from multiple sources.

**Results:**

The comparison exposes and contrasts two basic generic modes of commissioning:

1. Surrogate planning (English NHS), in which a negotiated order involving micro-commissioning, provider competition, financial incentives and penalties are the dominant media of commissioner power over providers.

2. Case-mix commissioning (Germany), in which managerial performance, an 'episode based' negotiated order and juridical controls appear the dominant media of commissioner power.

**Conclusions:**

Governments do not necessarily maximise commissioners' power over providers by implementing as many media of power as possible because these media interact, some complementing and others inhibiting each other. In particular, patient choice of provider inhibits commissioners' use of provider competition as a means of control.

## Health care commissioning as governance

How can commissioning be used for exercising governance over health-care providers in a quasi-market? To answer this question we compare the commissioning which has emerged from recent market-based reforms in England and Germany, analysing it in terms of the means that commissioners have for exercising power over providers. For England and Germany are archetypes of the Beveridge and the Bismarck systems of commissioning respectively, particularly for hospital services. Several other health systems structurally resemble the NHS (e.g. in Scandinavia) or are modelled on it (e.g. Italy, Spain, Portugal, Australia, New Zealand). The same applies to the German social health insurance (SHI) system of commissioning (e.g. France, Netherlands, Belgium, Switzerland, Russia).

### Health policy contexts

Most governments wish to maintain health care providers' accountability to the state. The 'bed-pan doctrine' often attributed [1] to Aneurin Bevan is a celebrated statement of this:

'if a bedpan is dropped in any hospital corridor, the noise should reverberate through the corridors of Whitehall' (p.103).

In quasi-markets, commissioners mediate the accountability chains [2] between government and providers. By 'commissioners' we mean organisations that select and/or finance other individuals and organisations to provide healthcare – whether social health insurers (e.g. Krankenkassen (Germany), Siekenfonds (Netherlands)), state bodies (e.g. Medicare (USA), National Commissioning Board (England)), groups of primary care doctors (e.g. Clinical Commissioning Groups (CCGs: England)), corporate insurers, charities or mutuals (e.g. Group Health (USA)). Many commissioners also, even mainly, act as the agents of employers, subscribers, shareholders and other interests.

Hitherto, NHS commissioning in England was conducted mainly by Primary Care Trusts (PCTs), centrally funded from taxation. For whole populations, typically of about 500,000, PCTs purchased hospital, community health and some primary medical care services by contracting a mixture of providers, some private but still mostly NHS-owned. A bare majority of general practices are commissioned through a national General Medical Services (GMS) contract whose implementation and payment PCTs monitor. PCTs are now being replaced by CCGs, membership organisations which are local networks of general practices. In many respects CCGs extend and develop the 1991 GP Fund-Holding scheme, for general practices will hold the budgets for their patients' non-GP primary care and most secondary care (see [3]). Access to non-emergency hospital care (which in England includes specialist ambulatory care) is solely by GP referral.

In Germany the main healthcare commissioners are SHIs ('sick-funds', Krankenkassen) and Land (provincial) governments. 87% of the population are SHI members (2012), the remainder privately insured or self-payers. Since 2007 the SHIs have competed for subscribers. Land governments plan the allocation of hospital beds and largely finance the corresponding infrastructure. Primary care doctors are commissioned by dividing a cash limited budget according to the points that each doctor has earned, with different numbers of points for different medical acts. Patients can self-refer to any 'ambulatory' doctor i.e. generalist family doctor or non-hospital specialist. Germany has long had a mixture of public (49% of beds in 2008), corporate (15%) and charitable (36%) hospitals.

Both Germany and England have introduced DRG-based systems (D-DRGs in Germany, HRGs or 'payment by results' in England) as the main method for hospital payment. The UK government is continues to promote corporate and third-sector provision of NHS-funded care; in 2008-9 only 7.1% of budgets was spent on non-NHS providers (general practices apart). Some English policy-makers hope that CCGs will compete for patients, but that has been a marginal theme in NHS reforms.

### Media of power

Commissioners generally try to control healthcare providers by using a combination of several methods in parallel [4]. We call each method of control a 'medium of power', because each embodies what Foucault [5] called a 'technology of power'. (He called a specific combination of methods a 'dispositif'.) Such combinations cannot be characterised a priori, only identified empirically [6]. Global overviews (e.g. [7,8]) suggest that healthcare commissioners generally use one or more of the following media of power.

#### Managerial performance of commissioning

In any quasi-market the managerial performance of commissioning consists essentially of:

1. Proposing contracts to potential providers and, where permitted, selecting providers.

2. Contract negotiation (see below)

3. Auditing and monitoring provider performance.

Transparency of provider activities and costs greatly assists commissioners in these activities [7].

#### Negotiated order

A negotiated order is an explicitly or tacitly agreed division of labour and concomitant rights of non-interference [9] between (here) commissioners and providers. Commissioners influence providers by influencing who participates in the negotiations, what topics are negotiable, and when they will be re-negotiated. The conduct and results of the negotiations depend partly upon the degree of trust between commissioners and providers i.e. whether the negotiators have similar or divergent interests, which in turn reflects the organisational aims, hence ownership and accountabilities, on either side. Where trust is strong a negotiated order centres upon discursive control (see below). The latter can still be applied even where trust is weak or absent, but then negotiators rely more on non-discursive mechanisms to align provider with commissioner interests artificially, including psychological pressure such as bullying in negotiations.

#### Discursive control

Commissioners have two discursive resources for persuading providers to comply with commissioner aims. Etic discourse (evidential, technical or scientific knowledge) nowadays means above all evidence based medicine (EBM) and epidemiology. As for emic discourse, i.e. discourse that is intelligible and morally persuasive to those who inhabit a particular culture but not necessarily to others, professions have what Foucault [5] called their 'discipline', supplementing technical knowledge with norms of conduct towards peers, superiors, clients and others; and wider ideologies (religion, economics etc.) [10]. The more that providers and commissioners speak the same discourses, the more persuasive one would expect a commissioner's arguments to be to a provider.

#### Financial incentives

For commissioners, the incentive effects of financial payments and penalties upon providers depends on:

1. how resource-dependent the provider is upon the commissioner

2. how much discretion the commissioner has to decide what services to finance, what unit of payment (per case, per diem etc.) the incentive or penalty attaches to [7], and to vary the payments or penalties accordingly.

These factors are not necessarily under the commissioners' or even providers' control. Commissioners in some health systems are restricted to reimbursing providers chosen by patients or GP gatekeepers. Then commissioners can only influence provider selection indirectly by framing ('nudging') the patients' or gatekeepers' choices [11].

#### Provider competition

Competition accentuates providers' resource-dependence. Commissioners' bargaining power depends on the number of organisations on either side of the market, with monopoly and monopsony as polar cases. Commissioners can strengthen their position by inviting new bidders, helping establish new providers, or providing services themselves ('make or buy' decisions). A commissioner facing a monopoly provider may nevertheless be able to use provider contestability – the credible threat of seeking or creating an alternative provider – to influence the provider [12]. Commissioners can also define the locus of competition i.e. criteria by which to select providers; at crudest, price versus non-price ('quality') competition.

#### Juridical governance

The legal system gives commissioners two control mechanisms over providers. One is how contract terms are specified, although there are always practical limits to contract completeness and presentiation [13]. The other is appeal to any regulative authorities (including those who license providers), special administrative courts (where they exist), the ordinary courts, or (in some systems) to higher governmental authority. Provider licensing and regulation are not usually commissioner prerogatives.

As noted, commissioners try to exercise governance over providers through particular combinations of the above media of power. We call each such combination a 'mode of commissioning'. Because its component media of power interact, each medium of power might be expected to have different effects even on similar providers (e.g. university hospitals) according to the institutional context in which it is applied. The structure of each quasi-market constrains which mode(s) of commissioning are available to commissioners within it. Hence market-based healthcare 'reforms' alter commissioners', and at one remove governments', capacity for governance over healthcare providers. The resulting differences in modes of commissioning therefore help explain among other things a health quasi-market's:

1. Patterns of provider development, including spread of medical technologies, the absence of specific kinds of provider or services, patterns of corporatisation and concentration of capital.

2. Capacity for cost control.

3. Patterns of managerial development of commissioning and medical involvement in it.

4. Development and use of evidence-based medicine.

This paper therefore examines how current post-reform modes of commissioning differ between the German and English health systems, and the implications for market-based 'reforms'.

## Methods

We systematically compared two national case studies (England, Germany) of current commissioning practice.

Data were collected at national, commissioner and provider level in Germany and England, 2011-12, by mixed methods from the following sources:

1. Interviews and discussions with key informants. In Germany these were representatives of the three the main federal associations of health organisations and the Federal Joint Committee (Gemeinsame Bundesausschuss (GB-A); 11 staff, covering a range of functions, one of the largest national SHIs; and managers from five hospitals (university, third-sector and publicly-owned). English interviewees, over 80 in all, came from PCTs, CCGs, Commissioning Support Groups, NHS hospitals and general practices.

2. Grey material including official regulations and guidance, for Germany, Sozialgesetzbuch V (SGB5) above all and for England the white paper [14] stating the rationale of the current commissioning system. .

3. Participation in three national events involving German SHIs.

4. Ad hoc enquiries from individual experts.

5. Published research found by hand-searching journals.

Interviews were recorded and transcribed. Material in German was translated either by two of the researchers or by native German speakers taking PhDs in England. The greater quantity of data collected in England reflects greater data collection there about differences in commissioning for different care-groups.

For systematic comparison the data were collated into an analytic framework structured according to the above media of power. Collating data from multiple sources provided an immediate means of triangulation, revealing gaps, ambiguities or apparent contradictions in the data, prompting supplementary data collection. The concept of power implies a counter-factual account of what providers would otherwise do [15] if commissioners were less powerful (and vice-versa), which we obtained empirically from informants' accounts of commissioners' attempts to change provider practice, of providers' response(s), and of what happened when providers proposed changes that the commissioners contested. We abstracted from the empirical comparison a characterisation of different generic modes of commissioning. Those differences and similarities which reflect the differences and similarities in quasi-market structures between the two countries are also likely to be relevant to other structurally similar quasi-markets. By comparing these generic modes of commissioning we drew some implications for health system reform policy, and corrected and refined our initial concepts of 'medium of power' and 'mode of commissioning' .

SW2 Research Ethics Committee gave ethical approval (09/H0206/50), conditional upon informant anonymity.

## Results

### Management of commissioning

#### Modelling versus planning

To launch the annual contract negotiations, German SHIs send each hospital spreadsheets of DRG targets. The proposed case-mix implies an overall number of DRG points, hence an implied budget. It is possible to reduce, even remove, groups of cases by reallocation within the total number of points but the system does not readily allow an overall reduction in case-load, case-mix or budget. National SHI federations, SHI national offices and local health-manager networks advise and update SHI negotiators about the current commissioning climate and local issues, but the hospitals have better data about their own case-mix and internal costs than the SHIs do. Continuously the SHIs audit, confirm and make payments, collecting from hospital bills and medical records data about what activity is being paid for. A Medical Review Board decides unclear or disputed cases; hospital staff tend to dislike its members.

English commissioners attempt to plan and finance all NHS service provision for a geographical population. Case-mix modelling is relevant to this activity but NHS commissioners usually predict demand en bloc on an historical basis, then decide how to finance new services and/or reallocate workload between providers. For small care-groups, commissioners often ally with other nearby commissioners to achieve managerial economies of scale and greater bargaining power when negotiating with specialised providers but, especially in larger cities, several NHS commissioners at times negotiate separately with one provider. An important difference is that NHS commissioning budgets are cash-limited but German SHIs can attempt to recruit more subscribers through marketing and offering more or better subscriber services (e.g. health promotion, information about available providers). In reality patient participation and marketing activities are marginal, if well-regarded, activities for NHS commissioners.

#### Provider transparency

The aforementioned activities give German SHIs strong information management systems. One SHI, reputedly the most developed in this respect, could routinely make such analyses as volumes of hip replacement revisions per provider and evaluation of disease management programme costs and outcomes for diabetes. SHIs compare their own data for hospital case-mix with the publicly available figures for all Germany, interrogating apparent inconsistencies between the two. Although, judging by what is publicly available, the range of nationally collected NHS data has increased substantially in the last three years, few NHS commissioners seem able to make similar analyses. The larger SHIs have economists, legal and other experts to advise (but not join) their local negotiating teams, in that way resembling Commissioning Support organisations in the NHS.

Nevertheless hospital activity is far from transparent to German SHIs. German hospital managers told us that when negotiating with commissioners they aggregated data and income data into large blocks:

I: *'Why did you decide [to do] that?'*

*Controllingschef: '[So that we need only] To make in all one negotiation about the DRGs and one for nursing care for children. And it's less transparent to the SHIs. … We have an orthopaedic department in [hospital 1] and one at [hospital 2]*, *and the SHIs could see from our data*, *our Excel tables*, *we have done this here but more there … if we handle matters at a large scale we don't have to discuss these things with the SHI'* (Hospital 4).

NHS providers' counterpart is a 'commercial - in confidence' argument for withholding information, although some PCTs reported an opposite problem, receiving masses of unanalysed data which left provider activity equally opaque. However the NHS incentive schemes (see below) which operate in parallel with HRGs expose some additional data, besides HRG out-turns, about hospitals' clinical work. Providers manipulating and misrepresenting activity data has become a problem in the NHS, incurring serious penalties. (Chief Executives have been sacked.)

### Negotiated order

#### Rhine versus principal-agent

In Germany, annual negotiations involving all main interest-groups (Federal associations of SHIs, doctors and dentists, hospitals and patient organisations), coordinated by the Gemeinsame Bundesausschuss and with the state as arbiter, set the broad framework of health service planning, including numerous guidelines for quality of care. (This 'Rhine' or 'Ordoliberal' approach is deeply rooted in German political culture [16]). They approve new treatments and diagnostic procedures for SHI reimbursement, the relative weights for the DRGs and how many points ambulatory care doctors are credited for each type of medical act. Although binding, these decisions are consensual. Consequently the doctors and SHIs have been able to block certain changes. For instance the Association of Ambulatory Physicians vetoed other doctors doing out-of-hospital surgical procedures [17]. The Euro value of the budget for dentists and ambulatory care doctors is negotiated between the Land doctors' chamber and the Land government, then at year end allocated retrospectively according to how many points each clinician earned. The Land can adjust the price of specific medical acts but not control the number or composition of ambulatory care providers. Similar negotiations establish a Land bed-plan based on predicted needs for hospital services. It frames the ensuing commissioner-provider negotiations by defining the each hospital's bed numbers, overall case-load, case-mix and, in effect, ceiling for SHI-funded activity. These negotiations focus on the hospital's DRG points allocation, case mix and the nationally-defined growth margin rather than clinical quality. The parties negotiate a 'corridor' for the main groups of DRGs, what rebates the SHI will receive should the volume or case-mix fall below that range and the payment for justified additional work above it. Generally the SHIs wish to avoid patient numbers, hence costs, spiralling out of control. Payment for new treatments not yet in the DRG system and for discretionary services are also negotiated.

Whilst the terms of GP contracts are negotiated nationally, the key negotiated order in the English NHS is established as each commissioner attempts to induce each of its providers to accept and implement the commissioner's plans for that provider's services; essentially a principal-agent negotiation [18]. It is typically – but not always – a bilateral negotiation with some flexibility as to when it occurs and what topics are negotiated. However, commissioners' agenda strongly reflects national policies, targets, priorities and guidelines. Hence the range of topics is typically wider and less precisely formulated than DRG-based allocations and performance measures alone. For service quality, the Care Quality and Innovation programme (CQUIN) now sets a range of specific targets, but depending on circumstances NICE or other guidelines can also be involved. (CQUIN sets quality standards which apply to all NHS hospitals. There are financial incentives for achieving them, and penalties for non-compliance.) The negotiations are framed by an annual contracting cycle although in practice non-marginal negotiations with a given provider may take place only every two or three years so as to reduce the managerial workload.

#### Episode-based versus micro commissioning

The main currencies of commissioning negotiations in Germany are for hospitals the number and case-mix of episodes and for ambulatory care doctors, medical acts. More than in Germany, NHS commissioners' negotiations with hospitals, community health services and mental health services also concern the processes of care, clinical quality of care, and the care pathways used, especially for types of care which are distributed over several separate providers (see [17,18]) and whose quality is hard to measure. NHS commissioners generally commission either a 'lead provider' to coordinate the others, or case managers. Either way the negotiated order (also) establishes arrangements for inter-provider coordination. This 'micro-commissioning' (as English GPs call it) contrasts with the focus on episodes of care in negotiations between SHIs and German providers; and indeed NHS commissioners' negotiations with corporate treatment centres. 'Relational contracting' in the NHS, at times almost adversarial, includes the informal exchange of information and the negotiation of adjustments to services and costs in between re-negotiations of a whole contract (see [13]).

### Discursive control

#### Emic discourse: solidarity versus authority

Informants in both countries mentioned emic discourses used in commissioning management and negotiations. German informants tended to refer to nationally negotiated agreements, themselves reflecting the 'solidaristic' Rhineland approach to policy formation mentioned above. In negotiations, they said, the regulations and different parties' rights and obligations regarding healthcare were often cited but these arguments could work both ways. When SHIs claim to represent patients' interests, the hospitals can reply that they – and SHIs – are equally obliged to ensure that patients can get the services that they (patients) choose. Apart from having to work within the Land bed-plan, considerations of public accountability did not appear to figure much.

Despite recent attempts to dilute it the English 'bed pan doctrine' remains strong. English law gives the Secretary of State for Health wide discretion, largely delegated to NHS commissioners who often justify particular commissioning proposals and decisions as being or implementing 'policy', ultimately the government's will. The politicisation and central control of NHS healthcare, through target-based performance management, is much more pronounced than in Germany. Our English informants at times also used new public management discourse, especially variants which represent markets and corporations as desirable models.

#### Evidential discourse

English informants made more frequent references to the use of EBM as a means of persuasion in commissioning negotiations than their German counterparts. Health technology assessments and NICE guidance play an increasing role in legitimating commissioning decisions in the NHS and in making clinical practice more transparent to non-medical managers. Provided they stick to treatments authorised under the Land plan and obey the law and regulations, German providers' treatment methods are beyond SHI scrutiny. The Medizinische Dienst der Krankenkassen (MDK) reviews patient case-notes in order to verify whether the coding and therefore payment were appropriate given the clinical facts, not to review effectiveness of care. In Germany the place of EBM is more at national level. When the GB-A decides which new therapies, devices, pharmaceuticals or models of care to include in the DRG tariff, they use above all evidence about effectiveness from the Institut für Qualität und Wirtschaftlichkeit in Gesundheitswesen (IQWiG). To recruit members, some SHIs pay for still-experimental treatments that they expect the D-DRG tariffs will eventually include, and for some complementary treatments having little evidence of efficacy. In contrast English PCTs and CCGs tightly control requests for such treatments, for example though special prioritisation committees.

### Incentives: pay for performance versus reimbursement

Both systems use pay-for-performance and reimbursement payments, but the NHS much more than the German system supplements tariff payments with pay for performance.

German commissioners mainly, and NHS commissioners partly, have to use predefined incentives which leave little local discretion for using financial incentives to reward providers for improving the clinical quality of care. In both systems, DRG-based payments give hospitals a financial incentive to increase activity. In Germany, only a few treatments (e.g. short-term nursing care at home) require prior SHI consent to pay. Since 1998 German SHIs have offered selective contracts, restricting subscribers' choice of providers in return for lower subscriptions. Patients must opt into these and into integrated care programmes, but many patients assume that only providers which have difficulty attracting patients will accept such contracts. For chronic care, the SHIs pay a per-diem Pflegekost and will from 2013 have the option to do the same (instead of making cost-plus payments) for psychiatric services. However the points tariff for ambulatory care has been modified to accommodate disease management programmes, i.e. preventive case management and continuous care for certain chronic conditions (e.g. diabetes, COPD). Some 14,000 such schemes exist but only about 5.5% of people are enrolled in them. The benefits appear to be improved care, at least for diabetics [21], rather than cost savings. Some experimental integrated care projects construct inter-organisational care pathways linking primary and secondary providers for certain patient groups, but they also require specially-negotiated contracts because DRGs aren't available for network-based care provision. Integrated care and disease management programmes represent only 1% of healthcare spending. Mostly, SHIs have to reimburse post facto whichever provider a patient chooses.

Within the GMS contract, NHS commissioners also have little discretion in setting financial incentives for general practices. The GMS contract predefines capitation and Quality and Outcome Framework (QOF) payments for service quality, the latter being arguably the most developed pay-for-performance incentive system in the healthcare world. Personal Medical Services (PMS) and Alternative Provider Medical Service (APMS) contracts are more flexible [22] if more laborious to manage. Nevertheless they do permit NHS commissioners to vary the financial incentives facing primary care providers and at times to reduce payments to them (more often for cost-saving than incentive reasons). NHS commissioners can also make ad hoc discretionary payments to general practices, for instance for additional services such as clinics for refugees. CCGs have inherited this mixture of incentive options, although recent press reports speculate that the different primary care contract types may soon be synthesised into a single format.

For hospitals, NHS commissioners supplement DRG-like payments with pay-for-performance incentives, often closely linked with policy imperatives. CQUIN incentives cut across specialties and are used to incentivise attempts to improve aspects of the quality of care (e.g. preventing hospital-acquired infection) which are not specific to one DRG. Commissioners of community health services have essentially the same range of incentives at their disposal. However, the NHS is now extending DRG-like payments to long-term (e.g. mental health) and end-of-life care, reducing commissioner latitude to determine incentives in these areas.

### Competition: any permitted provider

NHS commissioners can chose their providers. Except for selective and integrated care contracts, German commissioners cannot. Only the Land government can select or deselect hospitals as providers to be included in its bed-plan. For ambulatory care doctors and dentists, not even this mechanism is available. Any provider permitted under the plan and regulations is entitled to payment for whatever SHI-insured patients it can attract. Regulations intended to maximise provider diversity and competition for patients thus remove provider competition as a medium of power for the commissioners. The recently-announced UK policy of allowing 'any qualified provider' to treat NHS patients appears similar, although it remains to be seen whether NHS commissioners will have to pay such providers even in the absence of any prior contract with them.

Although the English GMS contract for general practices is negotiated at national level, CCGs can still prevent new general practices entering 'over-doctored areas' and use discretionary payments and APMS contracts to attract new primary care providers into under-doctored areas. A minority of primary care providers are now private firms working APMS contracts. The range of other primary care provision has widened considerably since 1998 to include community matrons (providing case-management for people with long-term conditions), GPs with special interests, walk-in centres, corporate general practices, nurse-led practices, hospital-based GPs and others. Wide though it is compared with German SHIs, NHS commissioners still do not use their discretion to select providers as much as English policy makers would like. Indeed when NHS commissioners appeared unwilling to engage corporate 'independent sector treatment centres' (now re-named 'NHS Treatment Centres'), the Department of Health commissioned them itself, with local commissioning bodies only subsequently implementing and monitoring the contracts [23]. Meantime the NHS has also introduced a policy of patient choice of hospital.

NHS hospitals were long constructed on a principle of one large hospital per district. NHS commissioners buy a mean of 66% of their secondary care from one hospital and another 22% from the next largest two. Hospital competition is limited, in practice, mainly to footloose services such as planned non-complex surgery. Germany, at least in the west, has a long tradition of multiple, diversely-owned hospitals in each locality. A great inhibitor of competition between English hospitals appears to be lack of hospital capacity compared with Germany, which in 2010 had 5.66 acute beds per 1000 population versus 2.37 in the UK ( <http://stats.oecd.org/index.aspx> accessed 17 Sept. 2012). This difference also results in German healthcare being less oriented towards primary medical care and community health services than the English NHS. As one SHI interviewee put it, ‘we have a hospital on every hill’.

### Juridical: administrative law versus half-written constitution

Much more than the NHS, German commissioning relies on nationally-standardised regulations, contracts and legal entitlements, clearly-specified decision-making processes and participants in them, and allocations of decision-making powers among particular institutions. The range and number of services offered, and remuneration rates, are stipulated at national level for all SHIs [24]. Until recently this system was used more to define specific entitlements and prohibitions to services and payments than to manage healthcare provision directly. German SHIs' obligations to patients are legally prescribed. They include a right to treatment by the patient's provider of choice (provided that the provider is eligible to be reimbursed), with the few exceptions noted above. In disputed cases a first step is to seek an independent opinion from MDK about the medical necessity of the treatment in question, followed by appeal to the Schiedstelle (administrative court) and then to the civil courts, but even Schiedstelle cases are infrequent (maybe one or two a year for the largest hospitals) and expensive (€7000 or more per case). The hospitals win perhaps 80% of these cases. Even in the juridically-oriented German system judicial remedies are for commissioners and providers alike instruments of last resort, used only exceptionally.

In contrast the UK juridical framework leaves the secretary of state considerable discretion, widened by the 2012 Health Act. NHS 'contracts' - i.e. agreements between two or more NHS bodies - are used almost as surrogate service planning documents. Although there is a standard national framework, NHS contracts appear less complete (less fully specified) than their German counterparts. The remaining NHS block contracts are less complete than 'payment by results' (HRG) specifications. Disputes about NHS contracts are resolved at the secretary of state's discretion not through the courts. NHS commissioning managers receive voluminous central 'guidance' but its legal and regulatory status is uncertain. However NHS commissioners and hospitals both know that Monitor (a national regulatory body) and regional level NHS bodies often intervene to 'help' the managers of NHS hospitals who fail to meet performance or budget targets, or whose services attract adverse publicity. NHS commissioners' contracts with non-NHS providers, including general practices, are more complete and presentiated. Only between NHS bodies and external providers – above all general practices – are contracts legally enforceable. One might therefore say that in a juridical sense the NHS has only a half-written constitution.

## Conclusion: modes of commissioning

This comparison focuses on services, excluding inter-sectoral public health, research, training, capital allocation and, above all, long-term care which in both countries is mostly financed separately (on England, see [24]). Our whole-system focus passes over more nuanced differences in commissioning between different care-groups (e.g. psychiatry versus heart disease) and localities. At the time of study CCGs were still developing. Many details and practicalities of their eventual work are so far unknown. The same applies to psychiatric commissioning in Germany. Comparisons with the non-English UK countries might yield different findings (see [13]). Below we compare just two main generic modes of commissioning. Others exist.

Nevertheless it is apparent that in both health systems the commissioners' managerial, negotiative and persuasive work reflects the commissioners' (differing) scope for discretion in choosing providers and the forms of incentive payments to providers, and behind them different regulative frameworks and property-relations in health care. In these respects the English and German health systems instantiate more generic modes of commissioning. Abstracting these generic patterns from the historical particularities of the two health systems, Table 1 summarises the similarities and differences between the different generic modes of commissioning which dominate the two health systems: case-mix commissioning in Germany, surrogate planning in the English NHS.

**Table 1:** Contrasting modes of commissioning

	Case-mix commissioning	Surrogate planning
Managerial Performance	Case-mix modelling + audit + subscriber marketing.	Service planning for geographical population.
Negotiated order	Consensus, multi-stakeholder model + episode-based.	Principal-agent + micro-commissioning
Discursive control	Emic solidarity + juridical rights + case-mix data.	Emic political authority + EBM
Financial incentives	Fixed tariffs.	Pay for performance (defined prospectively) + ad hoc + fixed tariffs.
Provider competition	None: referral 'framing' at most.	Competition or bilateral monopoly.
Juridical	Comprehensive regulation + administrative law.	Unwritten constitution + common law.

Under case-mix commissioning, managerial performance, negotiated order and juridical controls appear the dominant media of power. In surrogate planning, a different kind of negotiated order, provider competition and financial incentives and penalties dominate. Even where the same medium (e.g. negotiated order) is used in two health systems it may take different forms in each, depending on what other media of power co-exist and of course national political cultures.

Briefly returning to the aforementioned differences between health systems that differences in their modes of commissioning might help to explain, Table 1 suggests at least the following hypotheses:

1. The combination of a consensual negotiated order at national level, lack of provider competition (for commissioners' purposes) and rigid tariffs accommodates a diverse but arguably more stagnant mix of providers compared with commissioner selection of providers, flexible incentives and managerial disciplinary control. Björnberg, Garrofé and Lindblad [26] suggest that commissioning as surrogate planning, including micro-commissioning, together produce weaker performance on the Euro Health Consumer Index indicators (see http://www.healthpowerhouse.com/index.php?option=com_content&view=category&layout=blog&id=36&Itemid=55) than case-mix based commissioning does.

2. Regulations and tariffs which guarantee providers payment for all permitted treatments facilitate the spread of medical technologies and raise provider incomes, but thereby raise healthcare costs, compared with cash-limited commissioner budgets and service plans.

3. Case-mix management promotes the development of epidemiological and IT-based patterns of commissioning management. Micro-commissioning and clinician involvement in commissioning together promote more practitioner-focused and EBM-based patterns.

4. The combination of surrogate planning and micro-commissioning tends to promote EBM at provider level more readily than case-mix commissioning does.

Each of these hypotheses raises substantial future research questions.

## Discussion: limits of market-based reforms

Considering each mode of commissioning in the round with its interactions between the different media of power, the above comparisons suggest certain limits of market-based reforms. Provider diversification appears to generate a similar division of labour in either mode of commissioning. State-owned tertiary providers increasingly concentrate on high-complexity and hard-to-treat (e.g. multiple) conditions. The third sector also provides especially for the latter. Corporate providers specialise in profitable care, typically high-volume non-urgent acute care but (in Germany) also some more complex, high cost acute care (e.g. heart surgery). Tariff-based payment of competing providers appears on German experience to leave provider working practices less transparent than, say, negotiative order based on micro-commissioning which appears more readily to allow commissioners to promote evidence-based clinical practice and inter-organisational coordination of complex care.

If 'any qualified provider' comes to mean in the NHS what it does in Germany, it would prevent commissioners using selection (competition) of providers as a means of controlling them, despite – indeed, because of - patient choice of provider. Even if commissioners could use competition as a means of controlling providers, long term competition involving corporate providers appears, on German experience, to promote market concentration (mergers) on the provider side, weakening commissioners' scope for harnessing provider competition as a medium of control. Whether the patient or the commissioner chooses providers, only an excess of provision, creating the possibility of provider redundancy, results in providers competing for patients (or, in theory, commissioners). If in pursuit of 'austerity' governments constrain the supply of services they again weaken commissioners' power to harness competition between providers as a means of exercising governance over providers. If commissioners also cut their discretionary spending, that weakens or removes certain financial incentives too as means of controlling providers.

Government attempts to control the health system by regulation and tariffs (Germany) or by central, politicised control of commissioning and tariffs (English NHS) push provider competition into the marginal 'windows' [27] foreclosed by neither regulation, tariff nor policy fiat. Assuming that some degree of planning of the overall profile of healthcare provision is desirable, the German DRG system gives a concrete, detailed way of modelling and managing hospital activity, case-mix and revenue costs. It might be argued that deciding DRG volumes within the constraints of a Land bed-plan puts the infrastructural cart before the epidemiological horse of health care needs, whilst the NHS possesses the horse but not such a good cart. The contrast also suggests that the benefits of DRG-based service planning depend on an extensive IT infrastructure. DRG adoption alone is insufficient. When providers are entitled to payment once patients have chosen them, commissioners' control over provider costs is weakened from a budgetary cash-limited system (as NHS) into one which, at most, contains care costs within 'corridors'.

So commissioners' power over providers is not necessarily maximised by creating as many media of power as possible, for some media frustrate each other. A negotiated order complements juridical (contractual) control but – especially if it involves micro-commissioning - may simultaneously compromise competition. Tariff-based payments are not necessarily easy to reconcile with provider transparency and promoting EBM at provider level. Patient choice inhibits commissioners' use of competition as a means of controlling providers. Quasi-markets intrinsically separate consumer and payer, raising the question of whether providers compete for patients ('cash follows patients') or for commissioners' contracts (patients follow cash). Governments wishing to exercise power through the medium of commissioners choosing between competing healthcare providers cannot abdicate that choice entirely to patients.

## List of abbreviations used

APMS: Alternative Provider Medical Services [contract, England]; CCG: Clinical Commissioning Group; CQUIN: Care Quality and Innovation programme [England]; DRG: Diagnostic Related Group; D-DRG: Deutsche [German] DRG; EBM: Evidence-based medicine; EHCI: Euro Health Consumer Index; GB-A: Gemeinsame Bundesausschuss [Federal negotiating body, Germany]; GMS: General Medical Services [contract, England]; GP: General practitioner; HRG: Health Resource Group [English DRGs]; IQWIG: Institut für Qualität und Wirtschaftlichkeit in Gesundheitswesen [Institute for Health Care Quality and Economy, Germany]; MDK: Medizinische Dienst der Krankenkassen [Medical service for SHIs, Germany]; NHS: National Health Service [England]; NICE: National Institute for Health and Clinical Excellence; PCT: Primary Care Trust [England]; PMS: Personal Medical Services [contract, England]; QOF: Quality and Outcome Framework [of GP contracts, England]; SGB: Sozialgesetzbuch [Book of social law, Germany]; SHI: Social health insurance / insurer.

## Competing interests

The authors declare that they have no competing interests.

## Authors' contributions

All authors contributed to the overall structure of the paper and its analytic framework and approved the text. The first three authors undertook the German fieldwork. The first five undertook fieldwork in England.

## Declarations

Publication of this supplement was funded by the Nuffield Trust.

This article has been published as part of *BMC Health Services Research* Volume 13 Supplement 1, 2013: The Limits of Market-based Reforms. The full contents of the supplement are available online at http://www.biomedcentral.com/bmchealthservres/supplements/13/S1.
